# Anti-Inflammatory, Antioxidant, and Microbiota-Modulating Effects of Camellia Oil from *Camellia brevistyla* on Acetic Acid-Induced Colitis in Rats

**DOI:** 10.3390/antiox9010058

**Published:** 2020-01-08

**Authors:** Chun-Ching Wu, Yu-Tang Tung, Sheng-Yi Chen, Wei-Ting Lee, Hsin-Tang Lin, Gow-Chin Yen

**Affiliations:** 1 Department of Food Science and Biotechnology, National Chung Hsing University, 145 Xingda Road, Taichung 40227, Taiwan; ruby101012026@gmail.com (C.-C.W.); wilson1211983@gmail.com (S.-Y.C.); waitwaitwaiting@gmail.com (W.-T.L.); 2 Graduate Institute of Metabolism and Obesity Sciences, Taipei Medical University, 250 Wu-Hsing Street, Taipei 110, Taiwan; f91625059@tmu.edu.tw; 3 Graduate Institute of Food Safety, National Chung Hsing University, 145 Xingda Road, Taichung 40227, Taiwan; linhs@nchu.edu.tw

**Keywords:** camellia oil, *Camellia brevistyla*, antioxidant, anti-inflammation, gut microbiota, colitis

## Abstract

Altering the microbiota by the daily diet is highly associated with improved human health. Studies confirms the gastrointestinal protective and anti-inflammatory effects of camellia oil; however, the benefits in gut microbiota remain unclear. Camellia oils of *Camellia oleifera* (PCO) and *C. brevistyla* (TCCO) were used to evaluate probiotic growth in vitro. In addition, the protective effects of camellia oils in the acetic acid (AA)-induced colitis rat model were investigated. In vitro fermentation study showed the proliferation of *Lactobacillus* spp. and *Bifidobacterium* spp. from human intestinal microbiota was increased after TCCO treatment. Moreover, the rats pretreated with TCCO exhibited significantly less AA-induced colonic injury and hemorrhage, higher serum immunoglobulin G 1 (IgG 1) levels, lower malondialdehyde levels, and lower inflammatory cytokine production in the colon tissue compared with those in the PCO group. Surprising, the protective effect against acetic acid-induced colitis by TCCO was similar to sulfasalazine (positive control) treatment. Moreover, TCCO increased the richness and diversity of probiotics in gut microbiota. TCCO alleviated AA-induced colitis by modulating gut microbiota, reducing oxidative stress and suppressing inflammatory responses.

## 1. Introduction

Inflammatory bowel disease (IBD) is an ordinary chronic gastrointestinal tract inflammatory disease. However, the pathogenesis of IBD remains unclear. IBD includes Crohn’s disease (CD) and ulcerative colitis (UC), which show a high prevalence, of approximately 0.3%, in Western countries [1]. Although the etiology of UC is not exhaustively understood, a recent study reported that UC is associated with a decrease in antioxidant capacity and results in an increase in free radical and reactive oxygen species (ROS) production [2]. Colitis can lead to intestinal epithelial barrier malfunction and subsequently enhance the permeability of the barrier. Antigens enter the intestinal lumen through the permeable barrier and then attract lymphocytes and macrophages to accumulate for inflammatory factor and cytokine release [3]. Andoh et al. [4] indicated that the inhibition of inflammatory mediators can effectively slow IBD progression.

Both CD and UC activate innate (macrophage and neutrophil) and adaptive (T and B cell) immune responses, which cause diminished tolerance to enteric commensal bacteria [5]. Dysbiosis of the gut microbiota is common in IBD. Moreover, common drugs have incomplete efficacy with side-effects; therefore, finding a safe, effective, and economic alternative treatment strategy to prevent dysbiosis may be a new therapeutic strategy for IBD [6]. Previous studies have shown that camellia oil contains 2,5-bis-benzo [1,3]dioxol-5-yl-tetrahydro-furo [3–d][1,3]dioxine (named compound B) and sesamin that have high antioxidant activity [7], which could alleviate acute CCl_4_-induced liver damage in rats [8]. Camellia oil could also suppress the production of ROS and prevent nonsteroidal anti-inflammatory drugs (NSAIDs), such as ketoprofen and indomethacin, from causing intestinal mucosal damage [9]. In addition, camellia oil could alleviate ethanol-induced gastric damage [10] and significantly increase the relative abundance of *Bifidobacterium* and gut microbiota diversity [11]. Recent studies have shown that dietary habits are key factors that affect the composition of gut microbiota [12]. In this in vitro and in vivo study, we investigated whether camellia oils from *C. oleifera* and *C. brevistyla* can alleviate acetic acid (AA)-induced colitis by regulating the composition of the gut microbiota.

## 2. Materials and Methods

### 2.1. Preparation of Edible Oils

Camellia oils, PCO (*C. oleifera*) and TCCO (*C. brevistyla*), were provided by Dr. Ya-Lin Lee of the Taiwan Agricultural Research Institute. The Dorian extra virgin olive oil of Lakonia (OO) made in Greece) was purchased from Amway Taiwan Company (Taipei, Taiwan). Soybean oil (SO) were bought from Taiwan Sugar Corporation (Taipei, Taiwan). All the oil samples were kept in airtight in refrigerator at 4 °C until further use.

### 2.2. Chemical Features and Antioxidant Activity of Oil

The total antioxidant activity of edible oils was estimated by the Trolox equivalent antioxidant capacity (TEAC) assay according our previously report [7]. The total phenolic content and oxygen radical absorbance capacity (ORAC) of edible oils were determined as described by Yen et al. [13]. The α-tocopherol content in edible oils was assessed by HPLC and following the method of Flakelar et al. [14]. Fatty acid compositions were performed using Gas chromatography (GC).

### 2.3. In Vitro Gastric and Small Intestinal Digestion

In vitro gastric and small intestinal digestion was performed according to previously reported methods [15]. All of the digested samples were stored at −80 °C until further use.

### 2.4. In Vitro Fermentation of Human Intestinal Microbiota

The effects of camellia oils on human intestinal microbiota were explored by in vitro fermentation on the basis of a previously described method with few modifications [16]. Briefly, fresh fecal samples were collected from four healthy volunteers who had not received antibiotic or probiotic treatments during the preceding 3-month period and did not have gastrointestinal disorders. The volunteers were mentally and physically healthy and qualified for participation in the study. Each volunteer was informed and signed a consent form. A fecal slurry was prepared by stirring the fresh fecal samples with 0.1 M sterilized PBS (pH 7.2) to yield a 10% (*w/v*) suspension. Fermentation was initiated by adding 1 mL of the fecal slurry to 9 mL of basal nutrient medium (pH 7.0) containing 1 mg/mL digested samples and incubated anaerobically at 37 °C in an anaerobic jar. Next, samples were drawn from the inoculums at 0, 6, 12, and 24 h for bacterial computation (*Lactobacillus* spp., *Bifidobacterium* spp., and *C. perfringens*). All experiments were repeated more than three times.

### 2.5. Animal Grouping and Treatment Procedures

Six-week-old male Sprague–Dawley (SD) rats were purchased from BioLASCO Experimental Animal Center (Taipei, Taiwan) and given 1 week to accommodate to the environment. The protocols of animal experiments were approved by the Institutional Animal Care and Use Committee (IACUC) of National Chung Hsing University (Approval no: 104-130^R2^).

In this study, the beneficial effects of the edible oils were evaluated in rats with acetic acid (AA)-induced colitis. At first, 36 rats were randomly distributed into six groups (*n* = 6 per group). Colitis was induced in all groups by the transrectal treatment of 4% AA once on day 21, except for the SO group (denoted as the control subgroup). Experimental rat groupings: (1) SO group (daily oral gavage of 2 mL/kg BW soybean oil for 21 consecutive days); (2) AA group (daily oral gavage of 2 mL/kg BW soybean oil for 21 consecutive days. On day 21, transrectal administration of 2 mL of 4% AA); (3) SASP group as positive control group (daily oral gavage of 2 mL/kg BW soybean oil for 21 consecutive days. On day 21, transrectal administration of 2 mL of 4% AA and oral administration of 500 mg/kg BW sulfasalazine (Sigma-Aldrich Co. St. Louis, MO, USA) on days 21–24); (4) AA+PCO group (daily oral gavage of 2 mL/kg BW *C. oleifera* oil for 21 consecutive days. On day 21, transrectal administration of 2 mL of 4% AA); (5) AA+TCCO group (daily oral gavage of 2 mL/kg BW *C. brevistyla* oil for 21 consecutive days. On day 21, transrectal administration of 2 mL of 4% AA); and (6) AA+OO group (daily oral gavage of 2 mL/kg BW olive oil for 21 consecutive days. On day 21, transrectal administration of 2 mL of 4% AA). Fecal samples were stored on days 7, 14, 21, and 24 (after colitis establishment) for the enumeration of different bacteria (*Lactobacillus* spp., *Bifidobacterium* spp., and *C. perfringens*). Daily oral administration of edible oil was administered for 21 consecutive days. At the end of the experiment (day 24), rats were sacrificed, and the colon was immediately perfused with ice-cold 0.90% NaCl. All samples were stored at −80 °C for use in subsequent assays.

### 2.6. Bacterial DNA Purification and 16S rRNA Sequencing

On days 21 and 24 of treatment, fecal samples were collected from all the rats. After collection, the DNA was extracted using a QIAGEN DNA Mini Kit (Hilden, Germany). Furthermore, 16S rRNA sequencing was analyzed following our previous study [11], and the aforementioned bioinformatics analyses were conducted by Germark Biotechnology Co., Ltd. (Taihung, Taiwan).

### 2.7. Histopathology

For histological examinations, paraffin-embedded colon tissue sections were used. Hematoxylin–eosin (H&E) staining, histological injury score, and the degree of the lesion were performed according to the previously described method [8].

### 2.8. Preparation of Colon Homogenate and Total Protein Concentration

The colon tissue was homogenized, protein extraction was performed as previously described [9], and the total protein concentration of the colon tissue was measured with a commercial protein reagent kit (Bio-Rad, Hercules, CA, USA).

### 2.9. Measurement of IL-6, IL-1ß, and TNF-α Levels

Levels of interleukin-6 (IL-6), interleukin-1β (IL-1β), and tumor necrosis factor-α (TNF-α) in the colon homogenates were measured using monoclonal antibody-based enzyme-linked immunosorbent assay (ELISA) (Rat IL-6 DuoSet ELISA, Rat IL-1β DuoSet ELISA, and Rat TNF-α DuoSet ELISA; R&D, Minneapolis, MN, USA) as specified by the manufacturer.

### 2.10. Estimation of Superoxide Dismutase (SOD), Glutathione (GSH), Malondialdehyde–Thiobarbituric Acid Reactive Substances (MDA–TBARSs), and Myeloperoxidase (MPO)

The activities of SOD and MPO and the contents of GSH and MDA–TBARSs in the colon tissue homogenate were analyzed according to our previously described methods [9,10].

### 2.11. Statistical Analysis

The results are presented as the means ± standard deviation (SD) (*n* = 6). The significance of difference was calculated by Duncan’s test by using SPSS software, and results with *p* < 0.05 were considered to be statistically significant.

## 3. Results

### 3.1. Chemical Compositions and Antioxidant Activity of PCO and TCCO

The total phenolic contents were the highest in the TCCO group (17.3 mg of GAE/g extract), followed by the OO (16.4 mg of GAE/g extract), PCO (10.3 mg of GAE/g extract), and SO (8.3 mg of GAE/g extract) groups. PCO and TCCO have a fatty acid composition of oleic acid (802 mg/g of PCO and 788 mg/g of TCCO), linoleic acid (107 mg/g of PCO and 122 mg/g of TCCO), and palmitic acid (63 mg/g of PCO and 60 mg/g of TCCO). The ORAC of edible oils decreased in the following order: OO (150.8 mmol of Trolox/g extract) > TCCO (108.8 mmol of Trolox/g extract) > PCO (73.9 mmol of Trolox/g extract) > SO (27.0 mmol of Trolox/g extract). Meanwhile, for α-tocopherol, the results are as follows: SO (68.8 mg/100 g oil) > OO (25.8 mg/100 g oil) > PCO (20.6 mg/100 g oil) > TCCO (10.0 mg/100 g oil). The TEAC showed the highest amount for TCCO (1.4 μmol of Trolox/g extract), followed by PCO (1.3 μmol of Trolox/g extract), OO (1.1 μmol of Trolox/g extract), and SO (0.6 μmol of Trolox/g extract).

### 3.2. Effects of PCO and TCCO on the Bacterial Proliferation In Vitro

**Figure 1:**
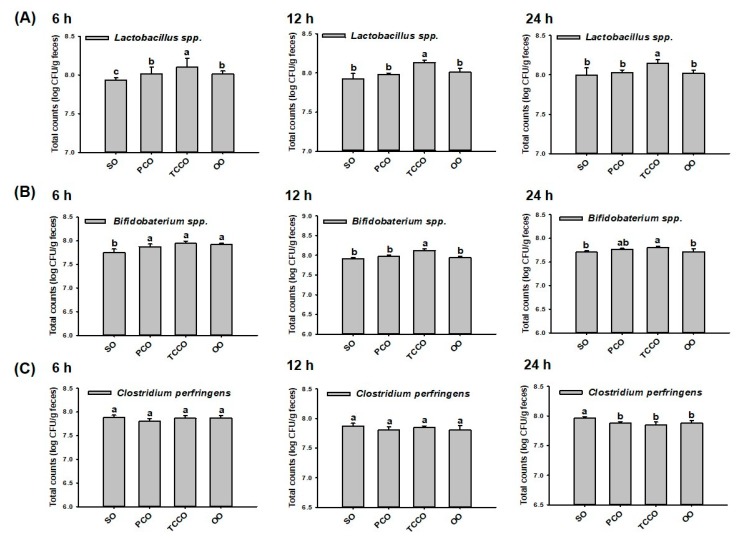
Gut microbiota (**A**) *Lactobacillus* spp., (**B**) *Bifidobacterium* spp., and (**C**) *Clostridium perfringens* in a batch-culture fermentation system containing SO, PCO, TCCO, and OO for in vitro fecal fermentation at 6 h, 12 h, and 24 h. The reported values are expressed as the mean ± standard deviation (SD) (*n* = 3). Values assigned different letters are significantly different at *p* < 0.05, which was determined using Duncan’s multiple range test. SO, soybean oil; PCO, camellia oil (*Camellia oleifera*); TCCO, camellia oil (*C. brevistyla*); and OO, olive oil.

At 0 h, the initial inoculum number of bacteria (*Lactobacillus* spp., *Bifidobacterium* spp., and *C. perfringens*) were at a concentration of 6.5 log CFU/g in all groups. At 6 h, the colony-forming unit (CFU) of *Lactobacillus* spp. in the TCCO group was significantly increased compared with the other groups (*p* < 0.05) (Figure 1A). Similar to the observation at 6 h, the CFU of *Lactobacillus* spp. in the TCCO group was significantly increased compared with the other groups at 12 h and 24 h (*p* < 0.05), respectively. These results indicated that camellia oil from *C. brevistyla*, namely, TCCO, exhibited a significant proliferation-promoting effect on *Lactobacillus* spp. At 6 h, the counts of *Bifidobacterium* spp. in the PCO, TCCO, and OO groups were significantly higher compared to the SO group (*p* < 0.05). At 12 h and 24 h, the bacterial growth rate of *Bifidobacterium* spp. in the TCCO group was significantly higher compared with the other groups (*p* < 0.05) (Figure 1B). These results indicated that TCCO exhibited a dramatic proliferation-enhancing effect on *Bifidobacterium* spp. The counts of *C. perfringens* differed nonsignificantly among all the groups at 6 and 12 h (Figure 1C). However, camellia oil treatment in the PCO, TCCO, and OO groups exhibited significantly lower counts of *C. perfringens* than did the SO group at 24 h.

### 3.3. Effect of Camellia Oils on the Growth of Gut Microbiota in Rats

**Figure 2:**
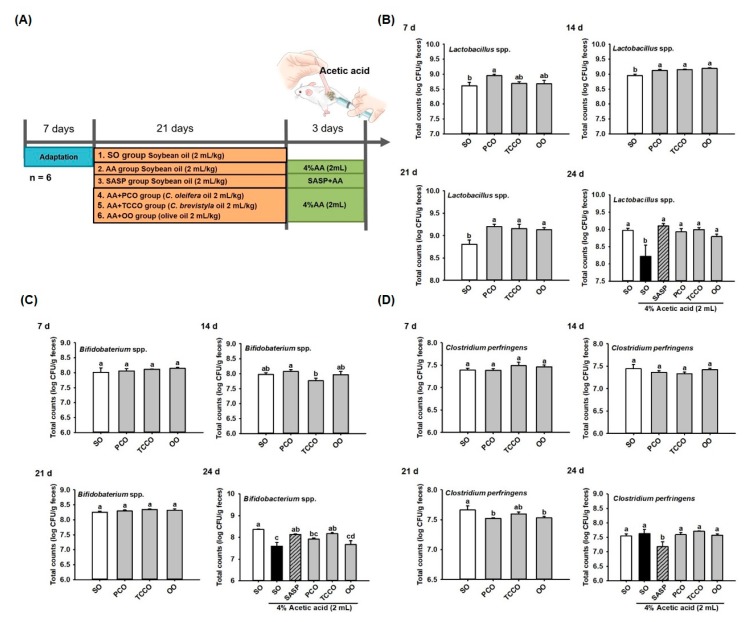
Effects of SO, PCO, TCCO, and OO on gut microbiota. (**A**) experimental groups, (**B**) *Lactobacillus* spp., (**C**) *Bifidobacterium* spp., and (**D**) *Clostridium perfringens* over 7, 14, 21, and 24 days in Sprague–Dawley rats. The rats received oral administrations of SO, PCO, TCCO, or OO for 3 weeks, and then, AA was administered intrarectally for colitis induction (except in the normal control group). SASP was used as a positive control after inducing colitis by using AA. The results are expressed as the mean ± standard error of the mean (SEM) (*n* = 6). Values assigned different letters are significantly different at *p* < 0.05, which was determined using Duncan’s multiple range test. SO, 2 mL/kg body weight (BW) of soybean oil; AA, 2 mL of 4% of AA; SASP, 500 mg/kg BW of sulfasalazine; PCO, 2 mL/kg BW of *C. oleifera* oil; TCCO, 2 mL/kg BW of *C. brevistyla* oil; and OO, 2 mL/kg BW of olive oil.

The experimental groups were illustrated in Figure 2A. Fresh fecal samples were collected on days 7, 14, 21, and 24 for bacteria (*Lactobacillus* spp., *Bifidobacterium* spp., and *C. perfringens*) enumeration from SO, PCO, TCCO, and OO-administered rats. On day 7, the *Lactobacillus* spp. count did not differ significantly among the groups. On days 14 and 21, the CFU of *Lactobacillus* spp. in the PCO, TCCO, and OO groups were greater than that in the SO group (*p* < 0.05). The counts of *Lactobacillus* spp. decreased significantly after colitis was induced using AA (*p* < 0.05). However, a higher total count of *Lactobacillus* spp. in the SASP, PCO, TCCO, and OO groups were observed compared to the SO group (*p* < 0.05) (Figure 2B). The counts of *Bifidobacterium* spp. after the rats received PCO, TCCO, and OO on days 7, 14, and 21 did not differ significantly from the count in the SO-fed rats. However, the count of *Bifidobacterium* spp. was significantly reduced after colitis was induced using AA (*p* < 0.05). Furthermore, the counts of *Bifidobacterium* spp. in the SASP and TCCO groups were significantly higher compared with the SO group on day 24 (*p* < 0.05) (Figure 2C). On day 21, lower CFU of *C. perfringens* in the PCO and OO groups were observed than that in the SO group (*p* < 0.05). After colitis induction, the fecal CFU of *C. perfringens* in the SASP group was significantly decreased compared with the AA-alone group (*p* < 0.05) (Figure 2D).

### 3.4. Effects of Camellia Oils on the Colon Length and the Levels of IgG1 and IgG2a

**Figure 3:**
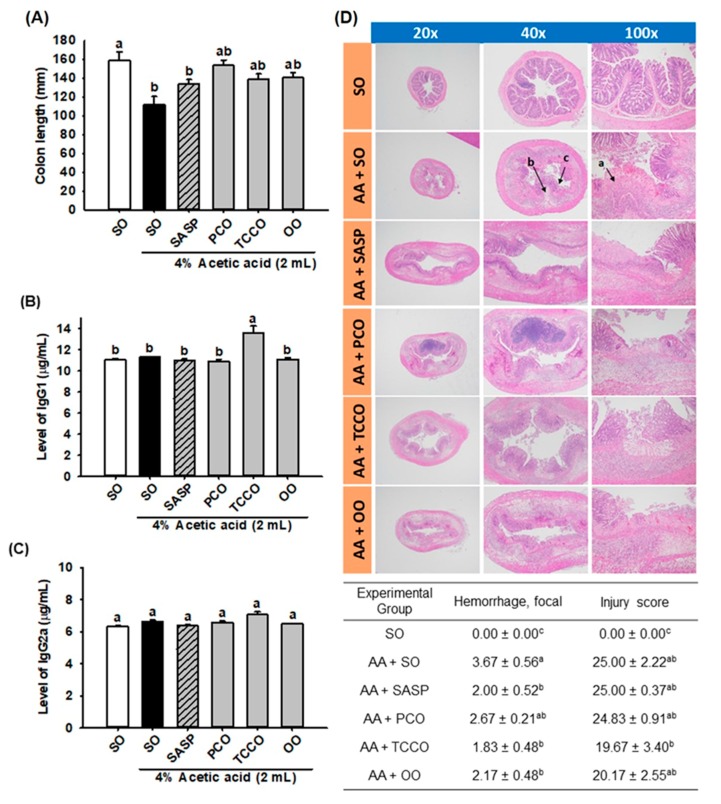
Effects of SO, PCO, TCCO, and OO on (**A**) colon length, (**B**) IgG1 levels, (**C**) IgG2a levels, and (**D**) hematoxylin and eosin (H&E) staining in Sprague–Dawley rats with AA-induced colitis. The rats received oral administration of SO, PCO, TCCO, or OO for 3 weeks. Then, AA was administered intrarectally for the induction of colitis (except in the normal control group). SASP was used as a positive control after inducing colitis by using AA. The rats were sacrificed on day 3, and their colon tissues were excised. The results are expressed as the mean ± SEM (*n* = 6). Values assigned different letters are significantly different at *p* < 0.05, which was determined using Duncan’s multiple range test. SO, 2 mL/kg body weight (BW) of soybean oil; AA, 2 mL of 4% of AA; SASP, 500 mg/kg BW of sulfasalazine; PCO, 2 mL/kg BW of *C. oleifera* oil; TCCO, 2 mL/kg BW of *C. brevistyla* oil; and OO, 2 mL/kg BW of olive oil. a, ulceration; b, edema; and c, lost crypts.

The length of the colons in the AA-treated group was shorter than that of the normal control group (*p* < 0.05) (Figure 3A). However, the colons in the SASP, PCO, TCCO, and OO groups were slightly longer than those in the AA-alone group. In addition, the TCCO group exhibited higher levels of IgG1 than the AA-treated rats (Figure 3B). Nevertheless, nonsignificant differences in IgG2a production levels were observed among the groups fed different oils (Figure 3C).

### 3.5. Effects of Camellia Oils on the Histological Changes in a Rat Model of AA-Induced Colitis

The results of the microscopic evaluation of the effects of camellia oils on the AA-treated rats are shown in Figure 3D. A regular colonic mucosa with the epithelium, crypts, and submucosa in the SO group was captured by microscopy. The AA-treated colitis group exhibited large regions of the colonic mucosa with ulcerations, edema, loss of epithelial or goblet cells, severe inflammatory-like cell infiltration, crypt architecture distortion, and crypt abscesses. SASP, TCCO, and OO treatments exhibited dramatic protective effects from AA-induced colitis by recovering the epithelium and crypts, inflammatory cell infiltration, and submucosal edema. The hemorrhage scores in the colitis groups treated with SASP, TCCO, and OO were lower than those in the AA-alone group. The injury score was dramatically reduced after TCCO uptake compared with the other groups, implying the powerful, potent antioxidant and anti-inflammatory abilities of TCCO.

### 3.6. Effects of Camellia Oils on Colonic MDA, MPO, SOD, and GSH Secretion in AA-Induced Colitis in Rats

**Figure 4:**
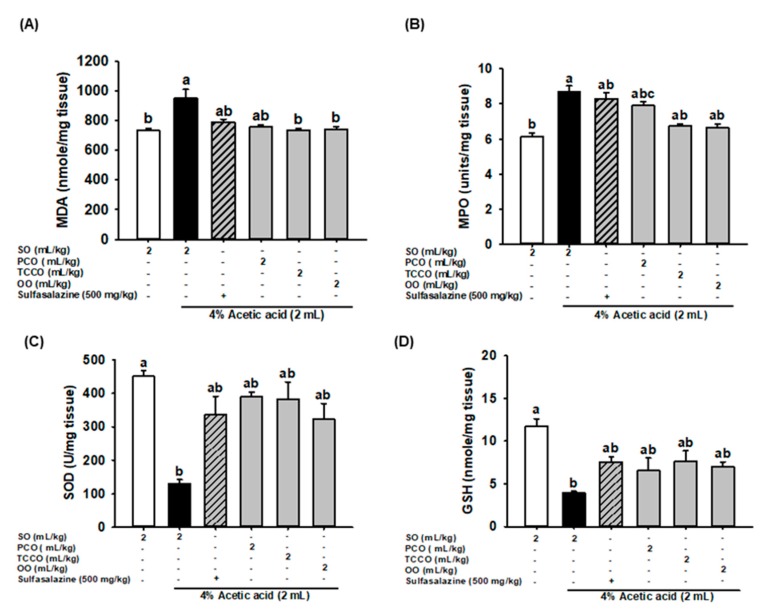
Effects of SO, PCO, TCCO, and OO on the levels of (**A**) MDA, (**B**) MPO, (**C**) SOD, and (**D**) GSH in the colonic mucosa of Sprague–Dawley rats with AA-induced colitis. The animals received oral administration of SO, PCO, TCCO, or OO for 3 weeks, and then, AA was administered intrarectally for induction of colitis (except in the normal control group). Sulfasalazine (SASP) was used as a positive control after colitis was induced using AA. The rats were sacrificed on day 3, and their colon tissues were excised. The results are expressed as the mean ± SEM (*n* = 6). Values assigned different letters are significantly different at *p* < 0.05, which was determined using Duncan’s multiple range test. SO, 2 mL/kg of body weight (BW) of soybean oil; AA, 2 mL of 4% of AA; SASP, 500 mg/kg BW of sulfasalazine; PCO, 2 mL/kg BW of *C. oleifera* oil; TCCO, 2 mL/kg BW of *C. brevistyla* oil; and OO, 2 mL/kg BW of olive oil. SOD, superoxide dismutase; MPO, myeloperoxidase; GSH, glutathione; MDA, malondialdehyde.

The effects of camellia oils on the production levels of colonic MDA, MPO, SOD, and GSH in the AA-treated rats are shown in Figure 4. AA treatment resulted in a significant increase in the levels of MDA and MPO and a decrease in SOD and GSH levels compared with the control group (*p* < 0.05). Pretreatment with TCCO and OO attenuated the increase in the MDA and MPO levels compared with the AA-alone group. However, pretreatment with SASP, PCO, TCCO, and OO slightly increased the levels of SOD and GSH compared with the levels in the AA-alone group.

### 3.7. Effects of Camellia Oils on Colonic IL-6, IL-1β, and TNF-α production in AA-Induced Colitis

**Figure 5:**
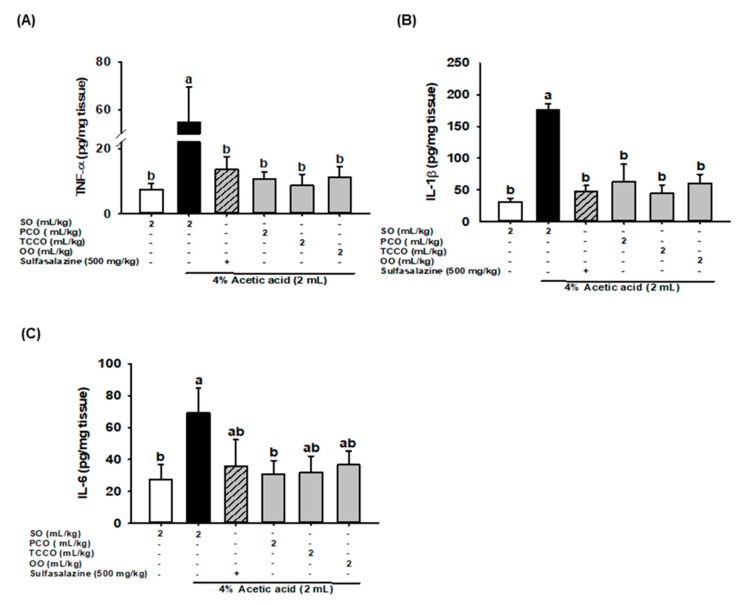
Effects of SO, PCO, TCCO, and OO on the levels of (**A**) TNF-α, (**B**) IL-1β, and (**C**) IL-6 in the colonic mucosa of Sprague–Dawley rats with AA-induced colitis. The rats received oral administration of SO, PCO, TCCO, or OO for 3 weeks. Subsequently, AA was administered intrarectally for the induction of colitis (except in the normal control group). SASP was used as a positive control after colitis was induced using AA. + (with SASP), − (without SASP). The rats were sacrificed on day 3, and their colon tissues were excised. The results are expressed as mean ± SEM (*n* = 6). Values assigned different letters are significantly different at *p* < 0.05, which was determined using Duncan’s multiple range test. SO, 2 mL/kg body weight (BW) of soybean oil; AA, 2 mL of 4% of AA; SASP, 500 mg/kg BW of sulfasalazine; PCO, 2 mL/kg BW of *C. oleifera* oil; TCCO, 2 mL/kg BW of *C. brevistyla* oil; and OO, 2 mL/kg BW of olive oil.

The effects of camellia oils on the alteration of proinflammatory cytokine levels, such as IL-1β, IL-6, and TNF-α, in the colonic tissue of the AA-induced rats are shown in Figure 5. The levels of IL-1β, IL-6, and TNF-α significantly increased after colitis was induced, and these levels were significantly reduced in the SASP, PCO, TCCO, and OO groups compared with the AA-alone group.

### 3.8. Effects of Camellia Oils on the Gut Microbiota of Rats

The composition of the gut microbiota in SO-, PCO-, TCCO-, and OO-treated rats were observed using the 16S rRNA gene. Significant changes in the gut microbiota were observed in the rats treated with different edible oils for 21 days. A Bray–Curtis dissimilarity analysis showed the richness and species diversity of the gut microbiota composition according to the operational taxonomic units (OTUs) method derived from clustering the 16S rRNA gene. The distributions of the gut microbiota in the TCCO and SO groups were different, which indicated that the composition of gut microbiota in the two groups were not similar. However, the distribution in the TCCO group was similar to the OO group, indicating that the composition of gut microbiota may be highly identical in the TCCO and OO groups.

**Figure 6:**
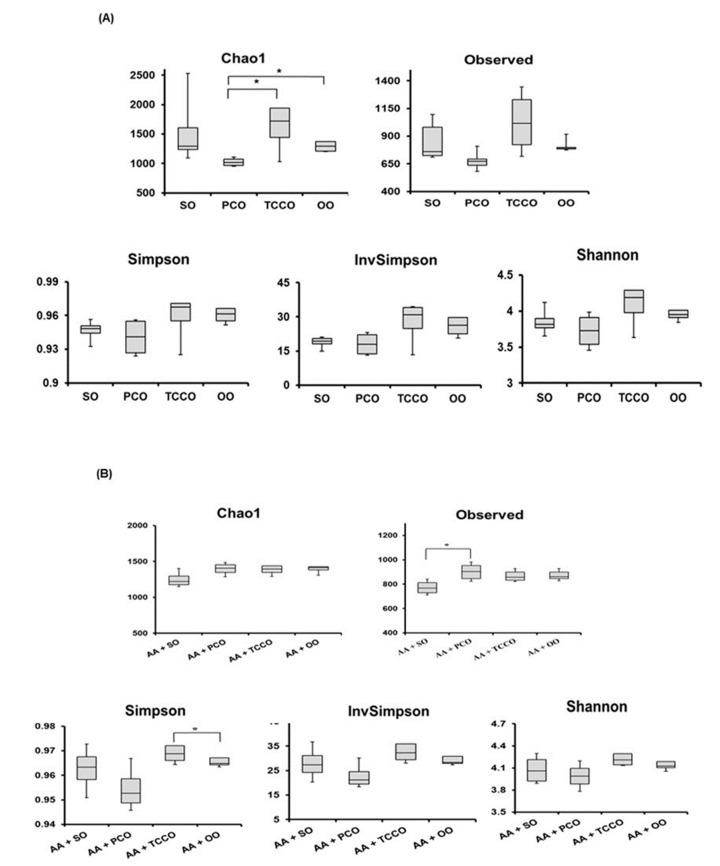

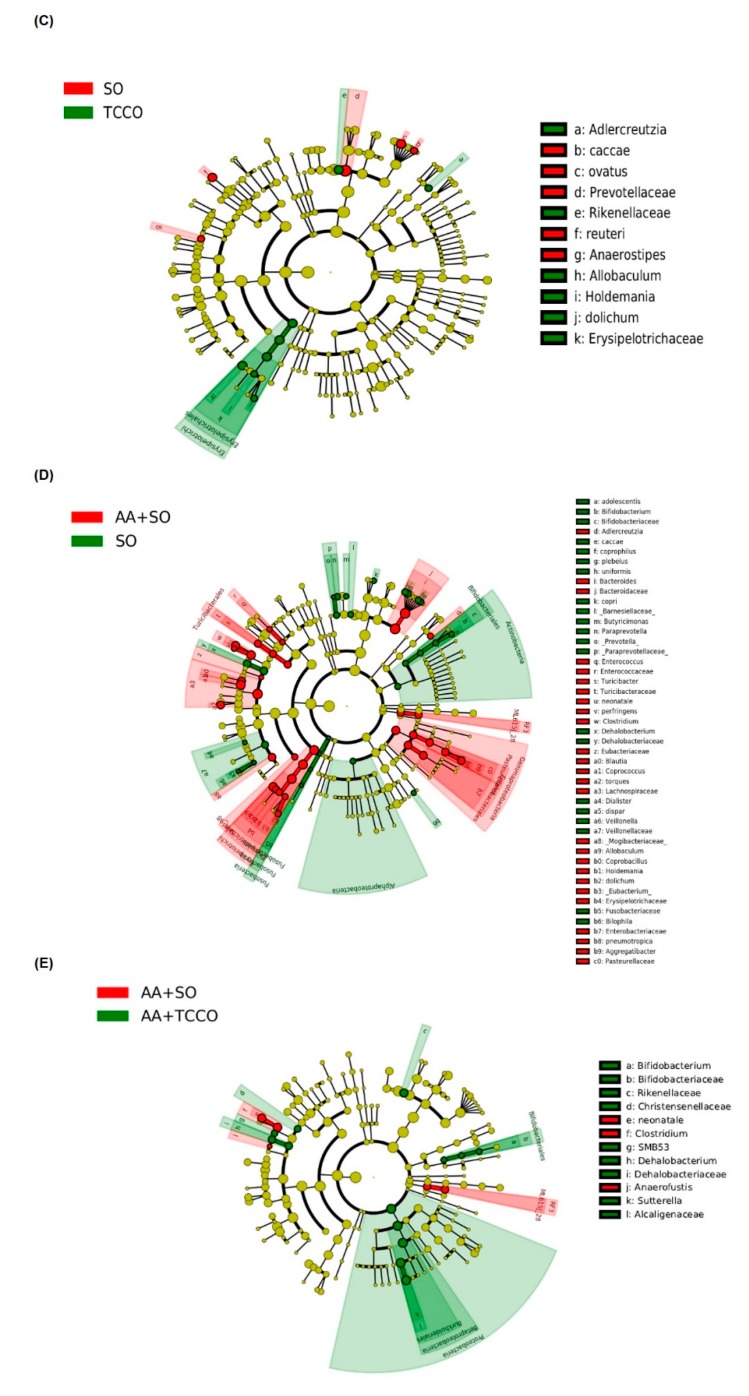
Alpha diversity indices of richness of gut microbiota composition in Sprague–Dawley rats fed SO, PCO, TCCO, or OO on days (**A**) 21 and (**B**) 24. The boxplot demonstrates the distribution summary of diversity indices estimated at the OUT level. (**C**) Cladogram generated from linear discriminant analysis (LDA) effect size (LEfSe) analysis, showing the most differently abundant taxa enriched in the microbiota obtained from the rats fed SO (red) or TCCO (green). The rats received an oral administration of TCCO or SO for 3 weeks. (**D**) LDA-LEfSe analysis, showing the most differently abundant taxa enriched in the microbiota from the rats fed AA + SO (red) or SO (green). The rats received oral administration of SO for 3 weeks. Subsequently, AA was administered intrarectally for induction of colitis, except for the normal control group. (**E**) LDA-LEfSe analysis, showing the most differentially abundant taxa enriched in the microbiota from SD rats fed AA + SO (red) or AA + TCCO (green). SO, 2 mL/kg BW of soybean oil; AA, 2 mL of 4% of acetic acid; and TCCO, 2 mL/kg BW of *C. brevistyla* oil (*n* = 4).

Alpha diversity was applied to analyze the complexity of species diversity for each sample. The richness of gut microbiota species communities usually presents in the Chao1 index and observed index, and the species diversity is usually estimated by the Shannon index [17]. The Chao1, Observed, Simpson, InvSimpson, and Shannon indices were higher in the TCCO-treated rats than in the other groups (Figure 6A). After colitis was induced using AA, the observed index increased significantly in the PCO-treated rats compared with the SO-treated rats (Figure 6B). In addition, the Chao1, Observed, Simpson, InvSimpson, and Shannon indices were slightly higher in the AA+TCCO group than in the AA+SO group.

As shown in Figure 6C, LEfSe (linear discriminant analysis (LDA) effect size) indicated that the counts of *Adlercreutzia*, Rikenellaceae, *Allobaculum*, *Holdemania*, dolichum, and Erysipelotrichaceae were higher in the TCCO-treated rats than in the SO-treated rats, whereas the counts of *Anaerostipes caccae*, *Anaerostipes ovatus*, Prevotellaceae, *Lactobacillus reuteri*, and *Anaerostipes* were higher in the SO-treated rats than in the TCCO-treated rats. AA treatment resulted in a decrease in the counts of *Bifidobacterium adolescentis*, Bifidobacteriaceae, Bifidobacteriaceae, coprophilus, plebeius, uniformis, copri, Barnesiellaceae, Butyricimonas, Paraprevotella, Prevotella, Paraprevotellaceae, and so on, as well as an increase in the counts of *Adlercreutzia*, *Bacteroides*, *Bacteroidaceae*, Enterococcus, and so on (Figure 6D). Figure 6E shows that after AA-induced colitis, the TCCO-treated rats exhibited higher counts of *Bifidobacterium*, Bifidobacteriaceae, Rikenellaceae, Christensenellaceae, SMB53, *Dehalobacterium*, Dehalobacteriaceae, *Sutterella*, and Alcaligenaceae than did the SO-treated rats, whereas the counts of *Clostridium neonatale*, *Clostridium* spp., and *Anaerofustis* were higher in the SO-treated rats than in the TCCO-treated rats.

## 4. Discussion

In this study, the human gastric and small intestinal digestion of edible oils and fermentation with human fecal microflora were modeled to analyze bacterial counts. Figure 1 shows that TCCO enhanced the counts of *Lactobacillus* spp. and *Bifidobacterium* spp. and reduced the count of *C. perfringens*. The anti-inflammatory, antioxidant, and composition of gut microbiota effects of camellia oils from *C. oleifera* and *C. brevistyla* were also evaluated in AA-induced colitis rats. The histopathological features and inflammatory mediators of AA-induced colitis are highly similar to those of human IBD. Furthermore, AA-induced colitis is commonly used to induce IBD because of its ease of use [18]. Injury caused by intrarectal injection of AA can lead to ulcers and inflammation in the inner wall of the colon. Microscopic images indicated that AA can cause crypt loss, ulcers, cell infiltration, submucosal edema, and hemorrhage in colonic tissue (Figure 3). Among the two camellia oils (PCO and TCCO), the TCCO-treated group exhibited notable retrieval of colonic mucosa injury from AA-induced colitis with a reduction in the submucosal edema and suppression of inflammatory cell infiltration. In addition, AA reduced the length of the colon (Figure 3A). Moreover, the PCO, TCCO and OO-treated groups slightly recovered the colon length. IgG, the highest in human serum, is synthesized in the spleen and lymph nodes. In rats, IgG is divided into 4 subtypes (IgG1, IgG2a, IgG2b, and IgG3), of which IgG1 and IgG2a are higher. Some induction mechanisms of colitis are related to the immune response [18], and thus, the IgG1 and IgG2a content was explored. Horton et al. [19] reported that low IgG1 levels adversely affect the progression of colitis, thus enhancing immunity as a treatment for colitis. Figure 3B,C show that the secretion of IgG2a did not significantly differ among all the groups, but the secretion of IgG1 in the TCCO-treated group was higher than that in the AA-alone group. Therefore, TCCO can enhance immunity and be used to treat colitis.

IBD causes an increase in the production of free radicals, such as the superoxide anion, hydrogen peroxide, hypochlorous acid, and hydroxyl radicals; thus, an increase in oxidative stress is a key causative factor of IBD. The animal model of AA-induced colitis causes an imbalance between the levels of free radicals and antioxidants, whereas neutrophil infiltration causes superoxide anion formation, and the production of ROS causes mucosal and tissue necrosis [20]. Camellia oils contain functional ingredients, such as flavonoids, catechins, and sesamin, which exhibit antioxidant effects [7,21]. In this study, camellia oil from *C. brevistyla* (TCCO) exhibited stronger antioxidant activity because of the higher ORAC and TEAC content and higher total phenolic content, but it also contained less α-tocopherol than camellia oil from *C. oleifera* (PCO). In addition, the results showed that TCCO treatment significantly reduced MDA levels as well as slightly reduced MPO levels and increased SOD activity and GSH levels. Therefore, TCCO treatment may increase the activity of antioxidant enzymes and slow oxidative stress in AA-induced colitis colon tissue. Moreover, we found the total phenolic contents, ORAC, and TEAC capacities between TCCO and OO are quite similar. The similar protective effects against acetic acid-induced colitis between AA+OO group and AA+TCCO group was also observed, indicating the potential of antioxidant for IBD treatment.

Colitis can cause intestinal epithelial barrier dysfunction and increase its permeability; furthermore, antigens can enter the intestinal lumen, causing the accumulation of lymphocytes and macrophages and resulting in the release of inflammatory factors and cytokines, such as TNF-α, IL-6, and IL-1β [3]. AA also induces the infiltration of neutrophilic white blood cells to release inflammatory cytokines, causing acute pathological changes in colonic tissues [11]. PCO and TCCO significantly reduced the secretion of TNF-α, IL-6, and IL-1β compared with AA alone (Figure 5). Thus, camellia oils (from *C. oleifera* and *C. brevistyla*) could reduce the secretion of inflammatory cytokines to retard the progression of AA-induced colitis.

Most polyphenols are present in food but are not absorbed in the upper gastrointestinal tract. Thus, the intestines are exposed to a considerable amount of unabsorbed phenolic substances, which can be used by the gut microbiota [7]. Polyphenols play a vital role with prebiotics in stimulating the growth of intestinal microbes (such as *Lactobacilli* and *Bifidobacteria*), inhibiting the growth of pathogenic bacteria, and contributing to the maintenance of intestinal health [7]. Xiao et al. [22] also reported that camellia oil is rich in oleic acid, polyphenolic compounds, and α-tocopherol, which can improve the richness and microbial diversity of *Bifidobacterium* in the intestine [11]. In this study, TCCO exhibited higher total phenolic content and linoleic acid, and it also contained less α-tocopherol, oleic acid, and palmitic acid than PCO.

In this study, rats were fed camellia oil for 24 days, and the bacterial counts in their feces were analyzed each week. Figure 2 shows that the TCCO-treated group had increased counts of *Lactobacillus* spp. and *Bifidobacterium* spp. and decreased counts of *C. perfringens*. Dicksved et al. [23] reported that probiotics (e.g., *Lactobacilli* and *Bifidobacteria*) could protect the intestinal mucosal barrier and immune system function, promote anti-inflammatory factors, and inhibit the growth of harmful intestinal bacteria. Camellia oils contain polyphenolic compounds such as catechins [10], which can significantly increase the counts of *Bifidobacterium* in the human intestine [24]. After the induction of colitis using AA, the counts of *Lactobacillus* spp. and *Bifidobacterium* spp. were significantly reduced. However, the PCO-and TCCO-treated groups exhibited a significant increase in the counts of *Lactobacillus* spp., whereas the TCCO-treated group exhibited a significantly higher count of *Bifidobacterium* spp. than that of the AA-alone group (Figure 2B,C).

Lee et al. [11] indicated that oil from *C. oleifera* increases the diversity and richness of gut microbiota. Therefore, in this study, we further explored whether different species of camellia oils (from *C. oleifera* and *C. brevistyla*) can retard the progression of AA-induced colitis by regulating gut microbiota.

The Chao1, Observed, Simpson, InvSimpson, and Shannon indices were higher in the TCCO-treated rats than in the other groups (Figure 6A). Among the rats with AA-induced colitis, the observed index was significantly higher for the PCO-treated rats than for the SO-treated rats (Figure 6B). In addition, the Chao1, Observed, Simpson, InvSimpson, and Shannon indices were slightly higher in the AA+TCCO group than in the AA+SO group. The maintenance of gut microbiota diversity can improve intestinal tract resistance to colitis [25]. Therefore, PCO and TCCO can protect the integrity and diversity of gut microbiota in AA-induced colitis in rats. Our previous study indicated that *C. oleifera* oil alters the composition of gut microbiota and improves AA-induced colitis in rats [11]. Thus, the composition of gut microbiota altered by *C. brevistyla* was explored in this study.

LDA-LEfSe indicated that the TCCO-treated group exhibited a higher relative abundance of Erysipelotrichales and Rikenellaceae than did the SO-treated group. Tao et al. [26] reported that an increase in the counts of Rikenellaceae provides enhanced intestinal protection. In addition, the SO-treated group exhibited a higher relative abundance in Prevotellaceae, and Darnaud et al. [27] showed that DSS-treated REG3A-TG mice (which are less sensitive to colitis) exhibited a lower level of Prevotellaceae than did DSS-treated normal mice. Therefore, higher counts of Prevotellaceae may increase the risk of colitis. Hansen et al. [28] reported that colitis changes the composition of gut microbiota. The AA+SO group had a relatively high abundance of Erysipelotrichaceae and Pasteurellaceae compared with the SO group. Kaakoush [29] indicated that Erysipelotrichaceae is associated with inflammation of the intestine and is highly abundant in patients with colorectal cancer. Glavan et al. [30] reported that an increase in Pasteurellaceae counts is associated with a decrease in the performance of pattern recognition receptors, which are involved in initiating innate immune responses.

The abundance of *Sutterella* spp. and Bifidobacteriaceae was relatively higher in the TCCO group than in the AA-alone group. *Sutterella* spp. is the main symbiotic bacteria present in the duodenum of healthy humans and can adhere to intestinal epithelial cells, playing a crucial role in immune regulation [31]. In the AA-alone group, the relative abundance of *C. neonatale* was higher than in the TCCO-treated group. Likewise, Roze et al. [32] indicated that *C. neonatale* is associated with necrotizing enterocolitis, and Sun et al. [33] showed that the richness of *C. neonatale* increased in patients with colorectal cancer. In conclusion, this study confirmed that among the two species of camellia (*C. oleifera* and *C. brevistyla*), oil from *C. brevistyla* is more effective in retarding AA-induced colitis by regulating the composition of gut microbiota, protecting the intestinal mucosa (in rats), and reducing the levels of induced oxidative stress and the inflammatory response. This finding might have a potential application for functional foods development.

## Abbreviations

AA	Acetic acid
CD	Crohn’s disease
CFU	Colony-forming unit
GSH	Glutathione
IBD	Inflammatory bowel disease
IBS	Irritable bowel syndrome
LDA	Linear discriminant analysis
LEfSe	LDA effect size
MDA	Malondialdehyde
MPO	Myeloperoxidase
NSAIDs	Non-steroidal anti-inflammatory drugs
OO	Olive oil
ORAC	Oxygen radical absorbance capacity
OUT	Operational taxonomic units
PCO	Camellia oil from *Camellia oleifera*
ROS	Reactive oxygen species
SASP	Sulfasalazine
SO	Soybean oil
SOD	Superoxide dismutase
TCCO	Camellia oil from Camellia brevistyla
TEAC	Trolox equivalent antioxidant capacity
UC	Ulcerative colitis
